# Clinical Cases

**Published:** 1889-05

**Authors:** C. N. Payne

**Affiliations:** Port Jervis, N. Y.


### CLINICAL CASES.

C. N. Payne, M. D., Port Jervis, N. Y.

Adelphia W., age eight years. Peritonitis. First saw patient January 4th, 1889, at ten P. M. She had been sick for about thirty-six hours, with the following symptoms: First twelve hours, more or less chilliness, but no history of distinct chill; sharp pains in lower abdomen, causing her to draw her limbs up and scream. Terrible "bearing down" pain on urinating. Bowels had moved three times, and had vomited twice green and yellow mucus.

Present condition: Temperature 101.5, pulse 120. Lying with limbs partially flexed. Abdomen extremely sensitive to least touch, even of bed-clothes, and pain aggravated by any movement or jarring. Pain very sharp, comes and goes quickly. Some distention of bowels. Rumbling of flatulence, etc. Severe pain on urinating, a "bearing down," but no smarting, burning, or cutting sensation. Urine scanty, but of normal appearance. Pain begins and is most severe in ilio-cœcal region, and extends down to bladder and then up on opposite side of abdomen. Inside surface of knees "black and blue," from striking them together during paroxysms of pain. But little thirst.

Patient's mother had given her Hyos., Acon., and one dose of Bell.$^{4}$, and then stopped it, as it seemed to aggravate all her symptoms. I prescribed Bell.$^{30}$ every fifteen minutes for four doses, and then every half-hour or hour.

At four P. M., summoned by telephone. Patient worse. Temperature 102.5. Mother said second dose of medicine aggravated, therefore had stopped it.

Intense throbbing headache, says "feels like hammers in her head." Eyes sensitive to light. Very sensitive to noise. Face very red and hot. Tongue, thin, white coating. More thirst. Cannot bear least touch of abdomen.

Prescribed Bell.$^{200}$, one dose (spoonful) after every paroxysm of pain.

At half-past nine P. M., temperature 102, pulse 116. Slight general improvement soon after taking last remedy, and had slept about two hours altogether, since last seen. Continued remedy.

January 5th, nine A. M., temperature 99.3, pulse 88, great improvement. Little or no headache. *Very little* sensitiveness of bowels; no distention of them. Scarcely any pain on urinating. Mother says, had considerable pain up to half-past two A. M., and some delirium, but had slept most of time after that hour until eight A. M.

At five P. M., found patient sitting in easy chair, dressed and able to walk about room without pain, in fact, well and very hungry.

