# Erratum

**Published:** 1889-06

**Authors:** 


Erratum.—In the May issue, page 199, second line, for *ten P. M.* read *two P. M.*

